# Organic Compounds in a Sub‐Antarctic Ice Core: A Potential Suite of Sea Ice Markers

**DOI:** 10.1029/2019GL084249

**Published:** 2019-08-27

**Authors:** A. C. F. King, E. R. Thomas, J. B. Pedro, B. Markle, M. Potocki, S. L. Jackson, E. Wolff, M. Kalberer

**Affiliations:** ^1^ British Antarctic Survey Cambridge UK; ^2^ Department of Chemistry University of Cambridge Cambridge UK; ^3^ Antarctic Climate and Ecosystems University of Tasmania Hobart Tasmania Australia; ^4^ Physics of Ice, Climate and Earth, Niels Bohr Institute University of Copenhagen Copenhagen Denmark; ^5^ Division of Geological and Planetary Sciences California Institute of Technology Pasadena CA USA; ^6^ Climate Change Institute University of Maine Orono ME USA; ^7^ School of Earth and Climate Sciences University of Maine Orono ME USA; ^8^ Now at: Research School of Earth Sciences Australian National University Canberra ACT Australia; ^9^ Department of Earth Sciences University of Cambridge Cambridge UK; ^10^ Department of Environmental Sciences University of Basel Basel Switzerland

**Keywords:** ice core, sea ice, sub‐Antarctic, biomarkers, Bouvet

## Abstract

Investigation of organic compounds in ice cores can potentially unlock a wealth of new information in these climate archives. We present results from the first ever ice core drilled on sub‐Antarctic island Bouvet, representing a climatologically important but understudied region. We analyze a suite of novel and more familiar organic compounds in the ice core, alongside commonly measured ions. Methanesulfonic acid shows a significant, positive correlation to winter sea ice concentration, as does a fatty acid compound, oleic acid. Both may be sourced from spring phytoplankton blooms, which are larger following greater sea ice extent in the preceding winter. Oxalate, formate, and acetate are positively correlated to sea ice concentration in summer, but sources of these require further investigation. This study demonstrates the potential application of organic compounds from the marine biosphere in generating multiproxy sea ice records, which is critical in improving our understanding of past sea ice changes.

## Key Points

- We analyzed a suite of organic compounds,alongside commonly measured ions, in the first ever shallow ice core from Bouvet Island
- Multiple marine‐sourced organic compounds are detected throughout the core
- Organic compounds, alongside MSA, show significant positive correlations with sea ice

## 1 Introduction

The use of organic compounds as biomarkers in ice cores holds great potential to yield new environmental proxies (Giorio et al., 2018). A very few organic compounds have been successfully quantified in ice cores and shown to be valuable tracers of past environmental conditions, for example, biomass burning markers such as levoglucosan and the sea ice marker methanesulfonic acid (MSA). However, atmospheric aerosol is up to 50% organic (Jimenez et al., 2009), leaving much remaining potential for application of organic compounds in ice core studies. In the sub‐Antarctic region palaeoclimate records are exceptionally sparse (PAGES 2k Consortium, 2017). Marine biogenic compound sources are prominent. Opening up a suite of new compounds in ice core analyses may significantly contribute to records of climate in the region.

Giorio et al. (2018) identified two groups of organic compounds which showed promise as marine‐sourced biomarkers in ice cores: fatty acids and secondary oxidation aerosols (SOA) of terpenes. A few existing studies find examples of these compounds in Greenland (Kawamura, Suzuki, et al., 1996) and Alaskan ice core records (Pokhrel, Kawamura, Ono, et al., 2015; Pokhrel, Kawamura, Seki, et al., 2015) over time scales of hundreds of years, with concentrations linked to both changing emission quantities and the strength and direction of atmospheric transport pathways.

Dicarboxylic and monocarboxylic acids are also important contributors to global marine aerosol; Fu et al. (2013) found that dicarboxylic acids contribute on average ∼15% to total marine organic aerosol. Compounds such as oxalate, formate, and acetate have been detected in remote locations along the coast of Antarctica in snow samples (Legrand & Saigne, 1988) and in the remote marine boundary layer in aerosol samples (Baboukas et al., 2000).

In the first ever ice core record from remote sub‐Antarctic island (SAI) Bouvet, we quantify the presence and interpret the records of an array of organic compounds alongside chemicals more commonly measured in ice cores (Table 1). Bouvet offers a chance to study a dominantly marine aerosol sourced location, without corresponding input of the same compounds sourced from the terrestrial biosphere. Thus, complexities in interpretation are reduced. These are the sole records of many of these compounds from the SAIs.

**Table 1:** Target Compound List for the Bouvet Ice Core, by Compound Group, With Ranges of Concentrations Detected in Bold

Source	Compound name	Formula	Concentrations this study (ppb)^a^	Method limit of detection (LOD; (ppb)
HPLC‐MS analyses (King et al., 2019)				
Isoprene SOA	Meso‐erythritol	C_4_H_10_O_4_	0.1–0.7^b^	3.1
Isoprene SOA	2‐Methyltetrols	C_5_H_12_O_4_	N.D.	0.6
Monoterpene SOA	Pimelic acid	C_7_H_12_O_4_	0.1–0.4^b^	0.1
Monoterpene SOA	1,2,4‐butanetricarboxylic acid (BTCA)	C_7_H_10_O_6_	N.D.	0.1
Monoterpene SOA	3‐methyl‐1,2,3‐butanetricarboxylic acid (MBTCA)	C_8_H_12_O_6_	N.D.	0.1
Monoterpene SOA	Terebic acid	C_7_H_10_O_4_	N.D.	0.1
Monoterpene SOA	Pinolic acid	C_10_H_18_O_3_	N.D.	0.1
Monoterpene SOA	*Cis‐*pinonic acid	C_10_H_16_O_3_	N.D.	1.0
Monoterpene SOA	Keto‐pinic acid	C_10_H_14_O_3_	N.D.	0.1
Sesquiterpene SOA	β‐caryophyllinic acid	C_14_H_22_O_4_	N.D.	0.1
Sesquiterpene SOA	β‐caryophyllonic acid	C_15_H_24_O_3_	N.D.	0.1
Sesquiterpene SOA	β‐nocaryophyllonic acid	C_14_H_22_O_4_	0.2–1.7^b^	0.3
Biomass burning	Levoglucosan	C_6_H_10_O_5_	N.D.	100
	D‐malic acid	C_4_H_6_O_5_	0.2–2.5^b^	0.1
	Salicylic acid	C_7_H_6_O_3_	N.D.	0.4
Marine/microbial: Low molecular weight fatty acids (LFA) (<C24)	Lauric acid	C_12_H_24_O_2_	N.D.	13.7
	Myristic acid	C_14_H_28_O_2_	N.D.	11.9
	Heptadecanoic acid	C_17_H_34_O_2_	N.D.	2.3
	Oleic acid	C_18_H_34_O_2_	2.4–7.6	2.1
	Nonadecanoic acid	C_19_H_38_O_2_	N.D.	0.3
	Arachidonic acid	C_20_H_32_O_2_	N.D.	0.1
	Behenic acid	C_22_H_44_O_2_	N.D.	0.3
	Tricosanoic acid	C_23_H_46_O_2_	N.D.	0.3
Terrestrial biomass: High molecular weight fatty acids (HFA) (>C24)	Heptacosanoic acid	C_27_H_54_O_2_	N.D.	0.5
	Octacosanoic acid	C_28_H_56_O_2_	N.D.	0.3
	Melissic acid	C_30_H_60_O_2_	N.D.	6.0
Ion chromotographic analyses (Littot et al., 2002; Mulvaney et al., 1992; Thomas & Abram, 2016)				
	Oxalic acid	C_2_H_2_O_4_	0.4–64.2	0.3
	Formic acid	CH_2_O_2_	12.61–1167.4	2.1
	Acetic acid	CH_3_COOH	6.6–1360.2	1.1
Dimethylsulfide (DMS)	Methanesulfonic acid (MSA)	CH_4_O_3_S	0.03–22.0	0.1
	Bromide	Br^−^	0.5–35.5	0.1
	Chloride	Cl^−^	149.5–7014.1	0.4
	Sulfate	SO^2−^_4_	0.1–903.4	0.1
	Nitrate	NO_3_^−^	7.1–1729.4	1.7
	Ammonium	NH_4_^+^	9.7–63.0	2.9
	Potassium	K^+^	8.4–329.9	0.6
	Sodium	Na^+^	77.8–4404.3	0.2
	Magnesium	Mg^+^	6.4–332.4	0.2
	Calcium	Ca^+^	9.3–608.2	1.7

*Note*. HPLC‐MS = high performance liquid chromatography mass spectrometry; SOA = secondary oxidation aerosols; N.D. = Not detected.

a Concentrations of compounds measured using the HPLC‐MS method are those after preconcentration of samples has been accounted for, thus some appear lower than the LODs.

b Discontinuous record.

## 2 Materials and Methods

### 2.1 Ice Core Site and Age Scale

Bouvet is a volcanic SAI at 3°E, 54°S. The Bouvet ice core was collected as part of the Sub‐Antarctic Ice Core Expedition project on board the Antarctic Circumnavigation Expedition cruise in 2017 (Walton & Thomas, 2018). The core was drilled on the eastern slope of the island at approximately 350‐m altitude using a Kovacs Mark II Coring System to a depth of 14.2 m. Ice was transported frozen back to Cambridge, UK, for later cutting and analyses. The core was dated using annual cycles in concentrations of oxygen isotopes and major ions, resulting in an age scale from 2001–2016 for the full core length (supporting information Figure S1).

### 2.2 Sample Preparation and Analyses

All ice core cutting was done using a steel band‐saw blade in a −25 °C cold lab at the British Antarctic Survey, UK. Ice core strips destined for fatty acid and SOA analyses were cut at annual resolution (January–December, based on the age scale determined in Figure S1). Samples were stored in precleaned glass vials. Annual samples were preconcentrated in a rotary evaporator preceding high performance liquid chromatography mass spectrometry analyses, following the methods of King et al. (2019). Analyses followed clean protocols for organics as described in King et al. (2019).

Separate ice core strips, destined for ion chromatographic analyses of major ions, MSA, oxalate (oxalic acid), formate (formic acid), and acetate (acetic acid), were cut and measured as discreet samples of 5‐cm resolution using a Dionex Integrion reagent‐free ion chromatograph in a class‐100 cleanroom (Littot et al., 2002; Mulvaney et al., 1992; Thomas & Abram, 2016). Plastic capped vials had been precleaned by microwaving in Milli‐Q water for 5 min and repeating three times. Values were later averaged to annual resolution.

### 2.3 Statistical Analyses

#### 2.3.1 Principal Components Analysis

Principal components analysis (PCA) with varimax rotation was carried out using annual average data for all compounds (Tables S1a, S1b, and S2) in the software RStudio (R version 3.5.1).

Input data were log transformed to normalize data distribution. Reliability of the initial PCA output was checked using a resampling method where repeat PCAs were run for the data set removing each compound from the matrix one at a time. The variance explained by each output component (PC1, PC2, etc.) was compared for each repeat run, determining whether one input compound is having a disproportionate impact on the output components. For the full data matrix PCA, principal components and variance explained were as follows: PC1 61%, PC2 18%, PC3 8%, PC4 6%, and all other components ≤2%; for all resampling PCAs, PC1 65–59%, PC2 20–15%, PC3 8–6%, PC4 6–4%, and all other components ≤2%. The low variation in PC% demonstrates no extremes in component values are caused by the removal of any one compound. Therefore, all are retained in the input data matrix.

Significance of components was decided based on Kaiser's Rule (Kaiser, 1960). The PCA retains the first four components as significant, explaining 93% total variance of the data. Varimax rotation was performed on these four significant components. PCA results are displayed in Table 2.

**Table 2:** Results of the PCA for Continuous Compound Records in the Bouvet Ice Core 2001–2016

Component:	PC1	PC2	PC3	PC4
Variance explained:	61%	18%	8%	6%
Ammonium	**0.549**	**0.774**	−0.130	
Nitrate		0.130	**0.974**	
Sulfate	**0.943**	0.118	−0.166	0.103
Sodium	**0.913**	0.195	0.170	0.257
Chloride	**0.924**	0.226	0.143	0.224
Potassium	**0.962**	0.108	−0.136	
Magnesium	**0.836**	0.414		0.251
Calcium	0.432	**0.690**		0.400
Oxalate	0.349	**0.886**	0.149	
Formate		**0.963**		0.192
Acetate		**0.943**	0.135	0.170
MSA	0.251	0.487		**0.787**
Oleic acid	**0.654**			**0.693**
Bromide	**0.906**	0.240	0.206	0.233

*Note*. Compounds contributing most strongly to each component are emphasized in bold. PCA = principal components analysis; PC = principal component; MSA = methanesulfonic acid.

#### 2.3.2 Sea Ice Correlations

We use two measures of sea ice conditions in this study. The first is Bootstrap (version 3.1) sea ice concentrations (SIC) from Nimbus‐7 scanning multichannel microwave radiometer and Defense Meteorological Satellite Program Special Sensor Microwave Imager‐Special Sensor Microwave Imager/Sounder. Data are available from the National Snow and Ice Data Centre (NSIDC) from 1979 onward (Comiso, 2017) and calculated as the percentage of ice cover within a 25‐km^2^ data cell. Spatial correlation plots are generated using NSIDC SIC in the Royal Netherlands Meteorological Institute (KNMI) Climate Explorer (https://climexp.knmi.nl/start.cgi). The concept here is to correlate a compound record over each grid cell in the region with the SIC of that corresponding grid cell, thus telling us where the ice core record obtained correlates with SIC. The correlation uses annual resolution compound time series and SIC for the 2001–2016 time period, calculating with detrended SIC data in 3‐month bins, one starting at each of the 12 months of the year. Temporal autocorrelation (intrinsic relationship between successive values of the same variable, artificially creating significant correlation) is accounted for by adjusting the number of degrees of freedom to account for lag‐1 autocorrelation in the data where degrees of freedom are larger than 1. Spatial autocorrelation (intrinsic relationship of SIC change between proximal grid cells) effects are discussed in section 3.2. Correlations presented in this study are the 3‐month averages found to have the strongest correlation between SIC and the respective compound record.

**Figure 1:**
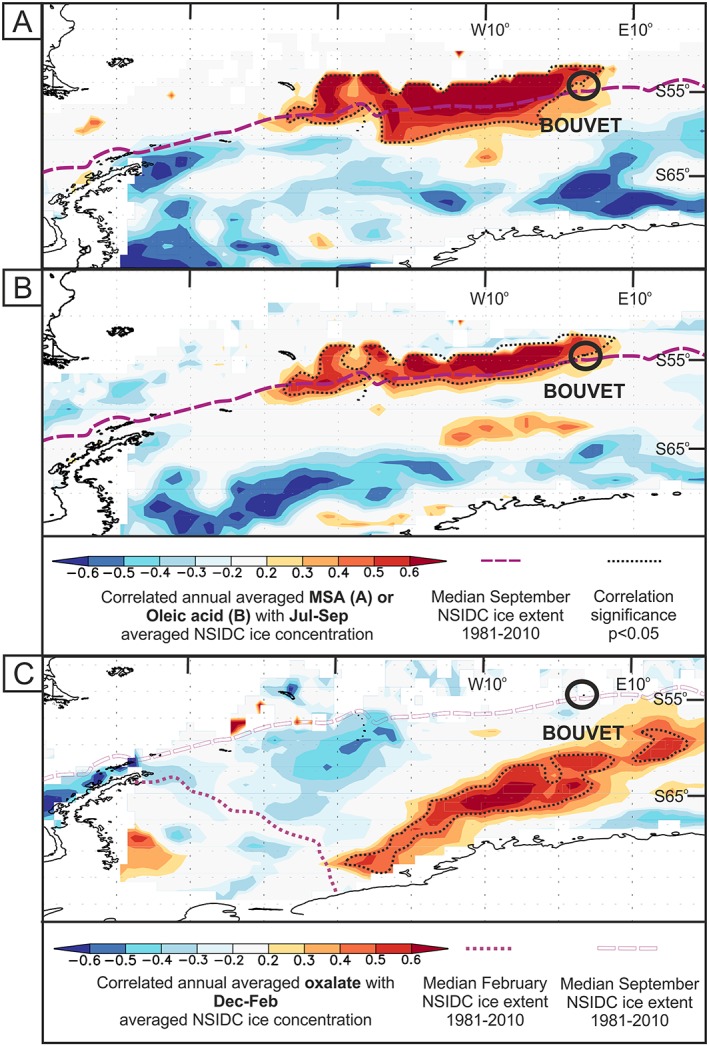
Maps of spatial correlation coefficients between (a) MSA, (b) oleic acid, and (c) oxalate with sea ice concentration in the region. Dotted black lines define areas of positive correlation significant at the *p* < 0.05 level. MSA = methanesulfonic acid; NSIDC = National Snow and Ice Data Centre.

Secondly, sea ice extent (SIE) is generated from the daily SIC data, calculating the area of all data points where sea ice was above a concentration threshold of 15% within the region. The median average SIE displayed in Figure 1 is a single‐line shape file from NSIDC for the period 1981–2010.

A geometric mean regression technique was used to calculate correlation coefficients between winter SIE (defined as the September average) and organic compound concentrations following the method of Abram et al. (2010) from Smith (2009). This accounts for measurement error in both SIE and compound data series (i.e., in the annual bulking of samples relying on precise dating and cutting, and analytical error from both sample preparation and instrumental analyses).

#### 2.3.3 Back Trajectory Modelling

The Numerical Atmospheric‐dispersion Modelling Environment back trajectory model was run for 1‐month periods for each of February and September in representative years 2008, 2009, and 2010. These months are those in which a compound had significant correlations to SIC (see section 3.2). The model was set to run 5‐day back trajectories based on the average of suggested atmospheric lifetimes for both fatty acids and SOA. (Donahue et al., 2013; Nozière et al., 2015; Rogge et al., 1991; Shi et al., 2002; Tsigaridis et al., 2014). Trajectories were run for each day of the month and later averaged to monthly periods (by averaging the values per grid cell for each output file).

## 3 Results and Discussion

Chloride, bromide, nitrate, sulfate, potassium, calcium, magnesium, ammonium, MSA, oxalate, formate, and acetate were all detected in significant concentrations in the Bouvet ice core (Table 1). Of the fatty acids and SOA components tested, a continuous record of just the fatty acid oleic acid was recorded. D‐malic acid, pimelic acid, meso‐erythritol, and β‐nocaryophyllonic acid were also detected; however, records were not continuous due to concentrations in several samples being below background contamination levels. This does not suggest background contamination was very high, but rather extremely low concentrations of the compounds present are sensitive to even small background contamination levels.

Many SOA compounds were not detected in any samples. Although isoprene and terpenes are known to have marine sources, they are minor in comparison to terrestrial sources (Hallquist et al., 2009). These results suggest terpenes do not have a significant enough marine source to be transported, deposited, and preserved in the ice at concentrations required for the limits of detection of the method used.

It is not surprising that long‐chain fatty acids (C27–C30), indicative of terrestrial sources (Eglinton & Hamilton, 1967; as opposed to shorter‐chain fatty acids (C < 27) indicative of marine sources), were not detected in the ice, this being such a remote marine location.

High background contamination is often an issue for fatty acid analyses as they are very common compounds in the natural environment. Although we follow contamination‐limiting protocols (as in King et al., 2019) all fatty acids except oleic acid were below background contamination levels. King et al. (2019) discuss possibilities of improving the analytical method used by tailoring the method solely toward fatty acids rather than the long list targeted in this study. With the promising results shown for oleic acid, further optimization of the method for this compound group is desirable.

### 3.1 PCA

Results of the PCA are displayed in Table 2. The component explaining most variance, PC1 (61%), shows strong contribution from the majority of compounds, especially major ions. This likely indicates that this is the transport component in the core, as transport is expected to contribute to the signal of most compounds to some extent. PC2 is the next strongest component (24%) dominated by the organics oxalate, formate, and acetate, and by ammonium and calcium. PC3 (8%) is dominated solely by nitrate, and PC4 (6%) by MSA and oleic acid.

There are two features of note in the organic compounds at this stage: the grouping of oxalate, formate, and acetate with ammonium and calcium in PC2 and the grouping of MSA and oleic acid in PC4. These associations are investigated further herein.

### 3.2 MSA and Oleic Acid

MSA is an often‐used sea ice marker (Curran & Jones, 2000, and references therein). The grouping of MSA and oleic acid in PC4 of the PCA was investigated by generating maps of spatial correlation coefficients of each compound to SIC in the region around Bouvet (Figures 1a and 1b).

MSA shows a significant region of positive correlation with SIC surrounding Bouvet at the end of winter and the initiation of spring (July–September), herein winter‐to‐spring. This region lies along the sea ice margin during maximum winter SIE and extends to the west along the sea ice margin. The correlation between oleic acid and SIC shows a very similar region of significance during the same period.

**Figure 2:**
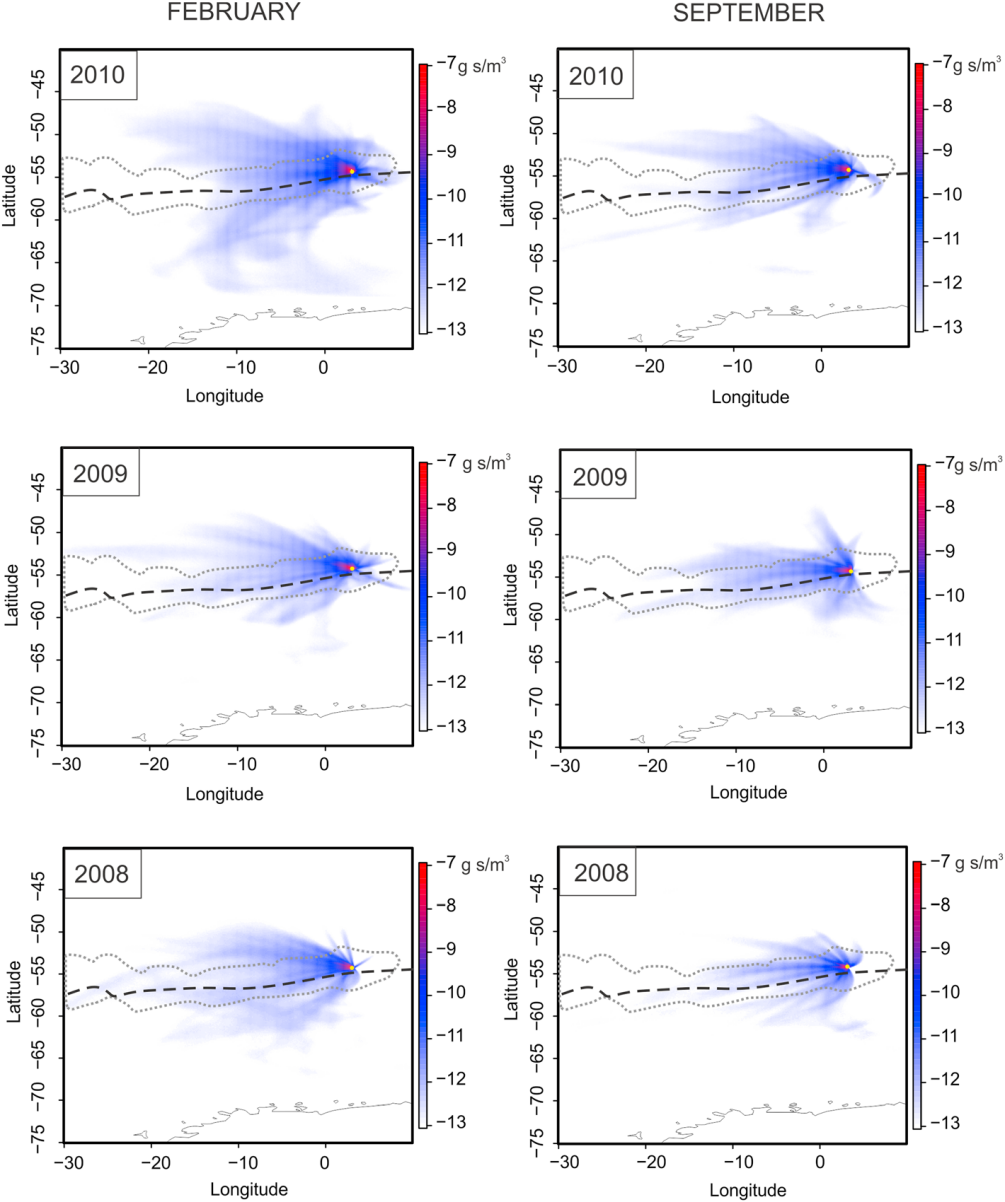
Example 1‐month averages of 5‐day back trajectories for each of February and September 2008–2010. Scales are log values. Plots include the outline of the oleic acid/sea ice concentration (SIC) correlation region as defined in Figure 1b.

The regions where we find substantial correlation of SIC to MSA and oleic acid lie along the median winter SIE, which is naturally a region of interannual variance in winter SIC. We should expect ~5% of the grid points on our correlation maps to exceed our significance test (*p* < 0.05) by chance and to do so in coherent regions owing to spatial autocorrelation in the sea ice variability. We find, however, that much greater than 5% of grid points are significantly correlated to our records. Further, the coherent regions of significant correlation are immediately adjacent to our site and almost solely up‐wind (as shown in the back trajectory analyses, Figure 2) providing a plausible physical relationship. Together this evidence suggests a mechanistic link between interannual variations in the summer SIC in the source region with MSA and oleic acid.

Considering mechanisms proposed in previous studies where MSA has shown a positive correlation to sea ice, a positive correlation is created by phytoplankton emitting precursor gases to MSA in large amounts along the sea ice margin, particularly during phytoplankton blooming events in sea ice break up during spring (Abram et al., 2013; Curran & Jones, 2000). Greater SIE during the winter leads to larger phytoplankton blooms in the following spring. These blooms may also enhance oleic acid concentrations. Fatty acids are dominant components of microalgae, and blooming events of diatoms and dinoflagellates have been shown to be associated with large increases in medium‐chain fatty acids in the water column, of which oleic acid was the dominant component (Kattner et al., 1983). Association between microalgae and oleic acid is so strong that the compound, alongside other fatty acids, may be used as class‐specific biomarkers of phytoplankton (Leveill et al., 1997; Sahu et al., 2013).

Supply of these compounds to Bouvet requires transport, which we investigate further using back trajectory analyses (Figure 2). A feature of all back trajectories is that transport is dominated by westerly winds, as is expected for the region. There is no significant difference observed between the February and September time plots within and between years. This consistent source region means that the chemistry recorded in the ice core is sampling the changes within that one region, aiding environmental interpretation of the time series.

Tracing the correlation region and SIE line over an example back trajectory plot (given for oleic acid in September 2010 of Figure 2) shows the region lies in agreement with transport from a source for both oleic acid and MSA along the sea ice margin to the west of Bouvet.

**Figure 3:**
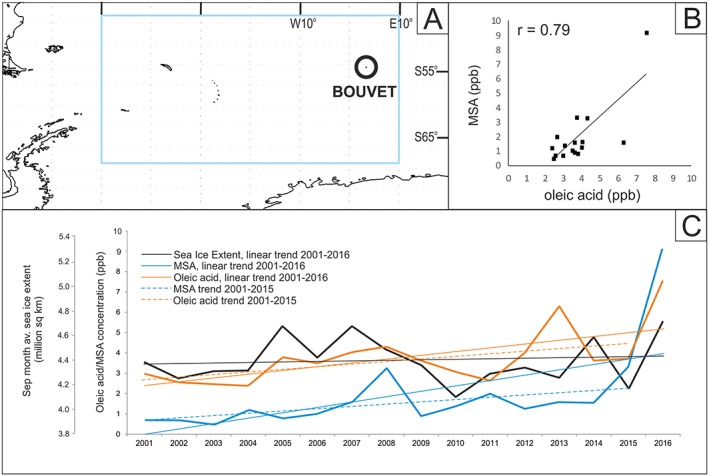
Oleic acid, MSA and maximum (September) sea ice extent time series with corresponding linear trends (c) for the region (a) 45–70°S, 50°W to 10°E. (b) Significant positive linear correlation between oleic acid and MSA, *r* = 0.79; *p* < 0.01. MSA and oleic acid are presented for two time periods (2001–2015 and 2001–2016) to account for any dominance of the trends by the high 2016 values, for which further description is found in section 3.2. MSA = methanesulfonic acid.

At Bouvet Island, a location on the edge of median maximum SIE, SIE variations may be particularly important in controlling phytoplankton blooms in the area. In years with lower ice extent, for example, the island may not actually be within the sea ice zone. To investigate this further, Figure 3 shows oleic acid, MSA, and maximum annual SIE in time series record for the entire length of the Bouvet core. The linear regression correlation coefficient for (nondetrended) oleic acid/MSA, *r* = 0.79 (*p* = <0.01), reinforces a link between the two compounds. Correlation coefficients between compounds and September SIE averaged over the 45–70°S, 50°W to 10°E area are *r* = 0.45 (*p* < 0.05) for oleic acid/SIE and *r* = 0.40 for MSA/SIE but *p* > 0.05, showing positive correlations with lower confidence in the case of MSA/SIE.

While the long‐term trend for SIE over the record 2001–2016 does not significantly increase or decrease, concentrations of both oleic acid and MSA show a slight increase. This may appear dominated by a sharp increase in concentration of both compounds in sample year 2016, but trend lines excluding this data point maintain a slightly reduced but still positive trend for 2001–2015. It has been shown previously that high diffusivity of MSA in ice samples may lead to a smearing of original concentrations of the compound deposited on the ice. However, this will not affect the record of compound variability along an ice core record if storage of samples has been consistent (Abram et al., 2008; Roberts et al., 2009). There is also no evidence of degradation of fatty acids down an ice core at least over time scales of a few hundred years in previous studies (Kawamura, Suzuki, et al., 1996). Therefore, it is likely that the overall trends in concentrations of MSA and oleic acid in the Bouvet ice core are representative of relative year‐to‐year values.

### 3.3 Oxalate, Formate, and Acetate

Oxalate, formate and acetate showed a strong association in PC2 of the PCA, alongside calcium and ammonium. The former compounds show a region of positive correlation to SIC, for which a representative example for oxalate is shown in Figure 1c. The strongest correlation period for oxalate was December–February. Correlation is naturally further south as SIE is lower (compared to the September MSA/Oleic correlation with SIC) during these months. Interpretation of this is challenging; the sources of these compounds in marine aerosol are not well investigated.

One previous study of snow and ice samples from coastal Antarctica attributed summer peaks in oxalate concentrations, alongside ammonium and calcium, to the presence of a large Ade1ie penguin population in the area from the end of October to March (Legrand & Saigne, 1988). The amount of sea ice along the coast each year would presumably affect the activities of penguin colonies. Back trajectories show organic compounds in the Bouvet core are unlikely to come from as far away as the coast of the Antarctic continent, where such colonies reside, within 5 days. However, much of the correlation area itself is outside the reach of most back trajectories suggesting the possibility that the material has survived longer transport times. Furthermore, the transport mechanism for these compounds may rely on not the air mass back trajectories but instead the travel of foraging penguins and sea birds from the sea ice margin toward Bouvet Island. These act as a direct input of these compounds on the island.

Alternatively, oxalate can form via degradation of a wide range of organic precursors, for example, fatty acids, emitted by phytoplankton to the atmosphere by sea spray processes (Kawamura, Kasukabe, et al., 1996; Miyazaki et al., 2010). In‐cloud oxidation of glyoxal has been observed as a specific route for oxalic acid formation in marine clouds (Rinaldi et al., 2011). As a result atmospheric photochemical oxidation is an important mechanism to consider in resulting concentrations of oxalate (Kawamura et al., 2001). The combination of phytoplankton productivity and photochemical degradation is likely to be strongest in summer, providing a source for our summertime oxalate correlation. This is however only speculative, and further studies of concentrations of these compounds in both marine aerosol and marine aerosol‐dominated ice cores will be beneficial in aiding interpretation.

## 4 Conclusions

A suite of marine‐sourced organic compounds have been detected in the sub‐Antarctic Bouvet Island ice core. MSA, a previously known sea ice marker, shows a positive correlation with SIC in the region of Bouvet during winter‐to‐spring. The region of significant positive correlation sits along the margin of maximum SIE and extends west from Bouvet due to dominant atmospheric transport from westerly winds. The correlation may be linked to phytoplankton blooming events which are larger when SIC in the region was greater during the preceding winter, occurring along the margin of initial sea ice breakup during the following spring. Strikingly, the organic compound oleic acid, a fatty acid constituent of phytoplankton, shows a strong positive correlation to MSA and indeed shows a positive correlation to SIC through the same spring period and geographical region, suggesting a potential new sea ice proxy.

Oxalate, formate, and acetate are found to have positive correlations with SIC in the later season of summer, and the correlation zone is further south than that of MSA and oleic acid. Further investigation is required to help identify the source of these compounds.

This study clearly highlights the application of organic compounds from ice core records as valuable marine biomarkers. The organic records here contribute not only to the data‐sparse sub‐Antarctic region but toward building a suite of sea ice proxies.

## Funding Sources

Work by Amy King was jointly supported by Selwyn College, Cambridge, and the NERC Doctoral Training Programme (Grant NE/L002507/1). ACE and Elizabeth Thomas received funding from École Polytechnique Fédérale de Lausanne, the Swiss Polar Institute, and Ferring Pharmaceuticals Inc. Joel Pedro acknowledges support from the European Research Council under the European Community's Seventh Framework Programme (FP7/2007e2013)/ERC Grant Agreement 610055 as part of the ice2ice project.

## Supporting information



Supporting Information S1

Table S1

Table S2

Table S3
