# Supplementary material for: Organic Compounds in a Sub‐Antarctic Ice Core: A Potential Suite of Sea Ice Markers
Source: Geophys Res Lett. 2019 Aug 27;46(16):9930–9. doi: 10.1029/2019GL084249 (PMC6853201; doi:10.1029/2019GL084249)
Supplement: Supplementary file 1 — Supporting Information S1 [file GRL-46-9930-s001.docx]

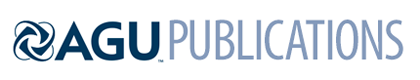


*Geophysical Research Letters*

Supporting Information for

**Organic compounds in a sub-Antarctic ice core: A potential suite of sea-ice markers**

A. C. F. King^1,2*^, E. R. Thomas^1*^, J. B. Pedro^3,4^, B. Markle^5^, M. Potocki^6,7^, S. L. Jackson^1†^, E. Wolff^8^, M. Kalberer^9^

^1^ British Antarctic Survey, High Cross, Madingley Road, Cambridge, CB3 0ET, UK.

^2^ Department of Chemistry, University of Cambridge, Lensfield Road, Cambridge, CB2 1EW, UK.

^3^ Antarctic Climate & Ecosystems, University of Tasmania, Hobart, Tasmania, Australia.

^4^ Physics of Ice, Climate and Earth, Niels Bohr Institute, University of Copenhagen, Copenhagen, Denmark.

^5^ Division of Geological and Planetary Sciences, Caltech, 1200 E California Blvd, Pasadena, CA 91125, USA.

^6^ Climate Change Institute, University of Maine, Orono, ME 04469, USA.

^7^ School of Earth and Climate Sciences, University of Maine, Orono, ME 04469, USA.

^8^ Department of Earth Sciences, University of Cambridge, Downing Street, Cambridge, CB2 3EQ, UK.

^9^ Department of Environmental Sciences, University of Basel, Klingelbergstrasse 27, Basel 4056, Switzerland.

^†^ Now at: Research School of Earth Sciences, Australian National University, Canberra, Australia.

**Contents of this file**

Figures S1

Tables S1a, S1b, S2

**Introduction**

This supporting information includes a figure showing the dating scale for the Bouvet ice core, and the annual resolution records for all cations, anions and organic compounds used in this study. Dating methods used can be found in the caption of Figure S1.


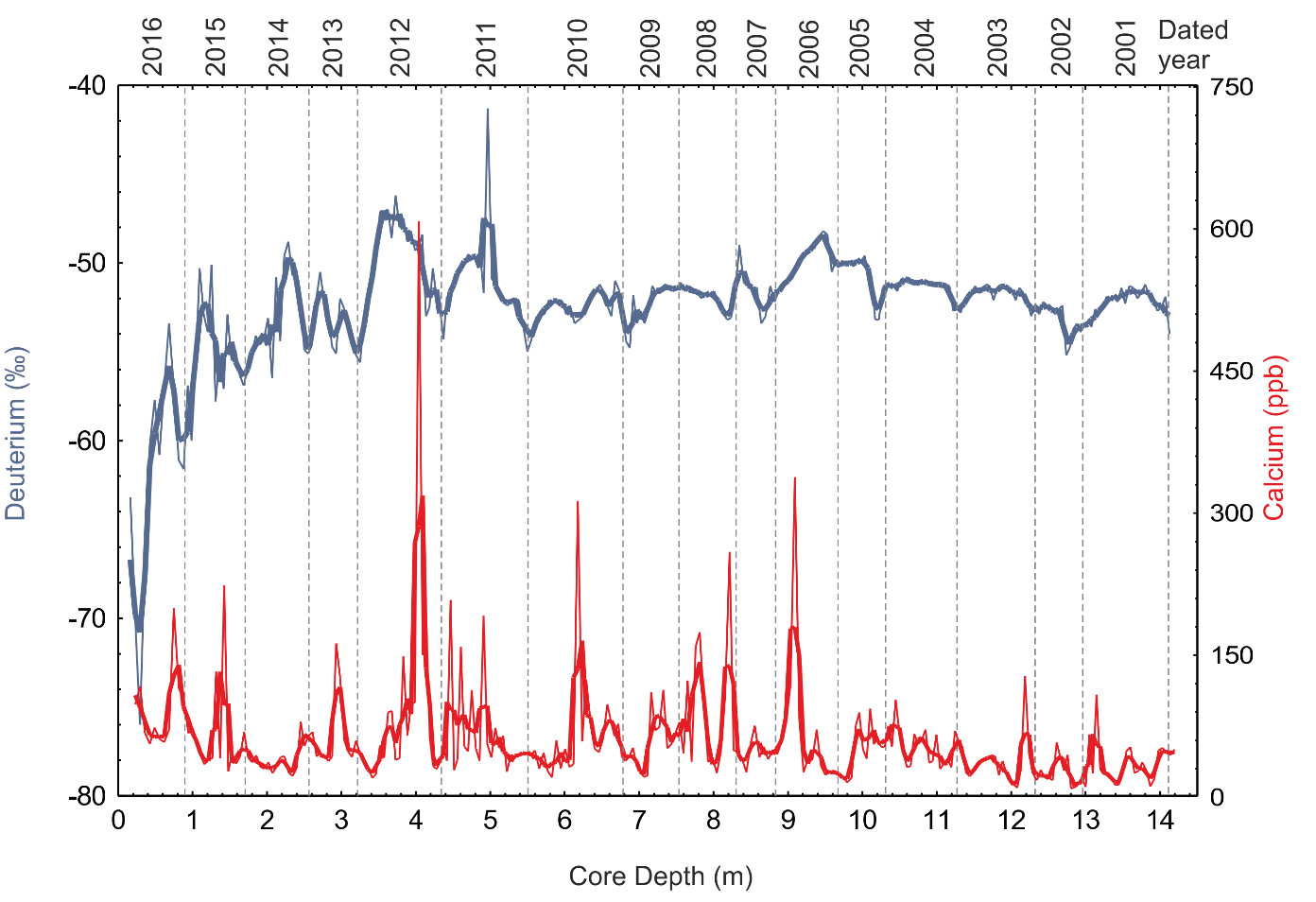


**Figure S1**: Layer counting method of dating the Bouvet record using deuterium isotopes, and where this was not clear, additionally using the calcium record which in some years did appear to show a summer-time peak. Each record had been smoothed over a three-point sliding data window to aid visual clarity in the plot. The years were dated winter-to-winter, based on annual minimum concentrations, to encompass summer-time peaks in concentrations in to one whole sample. This method resulted in an age scale from 2001-2016 for the full core length. Because of possible error in the judgement of annual layers based on the chemistry and isotope data, the validity of the layer counting method was checked using ERA5 data. ERA5 suggests a yearly average accumulation of approximately 0.65m weq (metres of water equivalent, representative of ice thickness and ice density). The final age scale for the Bouvet core results in an annual average of 0.59m weq.

**Table S1a:** Annual resolution concentrations (ppb) of major ions, used for PCA analysis.

**Table S1b:** Annual resolution concentrations (ppb) of major ions cntd. and oleic acid, used for PCA analysis.

**Table S2:** Annual resolution concentrations (ppb) of non-continuous organic compound records.
