# Supplementary material for: Organic Compounds in a Sub‐Antarctic Ice Core: A Potential Suite of Sea Ice Markers
Source: Geophys Res Lett. 2019 Aug 27;46(16):9930–9. doi: 10.1029/2019GL084249 (PMC6853201; doi:10.1029/2019GL084249)
Supplement: Supplementary file 2 — Table S1 [file GRL-46-9930-s002.docx]

|  | Compound | |  |  |  |  |  |  |
| --- | --- | --- | --- | --- | --- | --- | --- | --- |
| Year | Ammonium | Nitrate | Sulfate | Sodium | Chloride | Potassium | Magnesium | Calcium |
| 2016 | 26.0 | 46.4 | 194.7 | 1001.3 | 1659.8 | 48.8 | 80.7 | 91.0 |
| 2015 | 20.3 | 53.0 | 65.0 | 754.3 | 1187.8 | 38.4 | 38.8 | 62.0 |
| 2014 | 20.5 | 147.9 | 79.5 | 749.8 | 1168.7 | 33.3 | 52.5 | 42.2 |
| 2013 | 31.1 | 28.7 | 211.0 | 1221.6 | 1989.4 | 102.2 | 75.9 | 61.1 |
| 2012 | 38.9 | 54.0 | 173.6 | 1040.0 | 1695.7 | 60.8 | 71.3 | 101.5 |
| 2011 | 32.1 | 53.2 | 42.3 | 268.5 | 465.2 | 19.8 | 27.7 | 65.3 |
| 2010 | 29.2 | 46.0 | 124.0 | 533.9 | 998.9 | 44.8 | 47.3 | 68.5 |
| 2009 | 25.7 | 104.0 | 111.5 | 498.5 | 927.0 | 40.1 | 56.4 | 55.9 |
| 2008 | 35.0 | 47.0 | 65.2 | 300.8 | 561.1 | 27.4 | 52.2 | 84.8 |
| 2007 | 30.3 | 35.0 | 128.9 | 506.0 | 889.1 | 50.3 | 54.7 | 76.3 |
| 2006 | 20.2 | 22.9 | 86.6 | 342.9 | 601.4 | 27.0 | 36.7 | 39.2 |
| 2005 | 21.1 | 52.2 | 61.3 | 262.1 | 445.8 | 28.0 | 21.2 | 58.1 |
| 2004 | 24.7 | 39.3 | 79.3 | 295.5 | 547.5 | 33.1 | 26.7 | 35.8 |
| 2003 | 12.7 | 37.5 | 36.7 | 125.5 | 219.3 | 17.8 | 10.1 | 34.5 |
| 2002 | 15.2 | 39.6 | 34.9 | 140.7 | 257.3 | 18.1 | 15.3 | 31.4 |
| 2001 | 25.7 | 29.7 | 123.3 | 431.7 | 812.9 | 43.7 | 37.6 | 37.2 |
