# Supplementary material for: Organic Compounds in a Sub‐Antarctic Ice Core: A Potential Suite of Sea Ice Markers
Source: Geophys Res Lett. 2019 Aug 27;46(16):9930–9. doi: 10.1029/2019GL084249 (PMC6853201; doi:10.1029/2019GL084249)
Supplement: Supplementary file 3 — Table S2 [file GRL-46-9930-s003.docx]

|  | Compound | |  |  |  |  |
| --- | --- | --- | --- | --- | --- | --- |
| Year | Oxalate | Formate | Acetate | MSA | Bromide | Oleic acid |
| 2016 | 10.5 | 93.9 | 123.0 | 9.2 | 6.5 | 7.6 |
| 2015 | 14.6 | 110.0 | 165.7 | 3.4 | 4.6 | 3.7 |
| 2014 | 7.3 | 47.9 | 40.1 | 1.6 | 5.0 | 3.6 |
| 2013 | 8.5 | 42.0 | 32.4 | 1.6 | 8.6 | 6.3 |
| 2012 | 15.8 | 123.9 | 200.8 | 1.3 | 7.2 | 4.0 |
| 2011 | 15.7 | 138.6 | 187.4 | 2.0 | 2.0 | 2.6 |
| 2010 | 12.7 | 121.6 | 199.2 | 1.4 | 3.6 | 3.1 |
| 2009 | 9.2 | 71.2 | 93.4 | 0.9 | 4.0 | 3.6 |
| 2008 | 23.3 | 286.7 | 412.8 | 3.3 | 2.1 | 4.3 |
| 2007 | 12.9 | 109.6 | 57.0 | 1.7 | 3.4 | 4.0 |
| 2006 | 2.8 | 35.9 | 37.1 | 1.1 | 2.0 | 3.5 |
| 2005 | 4.5 | 36.8 | 50.4 | 0.8 | 1.5 | 3.8 |
| 2004 | 8.6 | 54.8 | 70.8 | 1.2 | 1.6 | 2.4 |
| 2003 | 1.1 | 22.0 | 13.3 | 0.5 | 0.8 | 2.5 |
| 2002 | 4.6 | 50.6 | 54.8 | 0.7 | 0.9 | 2.5 |
| 2001 | 6.7 | 47.0 | 40.8 | 0.7 | 2.7 | 3.0 |
