# Supplementary material for: Organic Compounds in a Sub‐Antarctic Ice Core: A Potential Suite of Sea Ice Markers
Source: Geophys Res Lett. 2019 Aug 27;46(16):9930–9. doi: 10.1029/2019GL084249 (PMC6853201; doi:10.1029/2019GL084249)
Supplement: Supplementary file 4 — Table S3 [file GRL-46-9930-s004.docx]

|  | Compound |  |  |  |
| --- | --- | --- | --- | --- |
| Year | D-malic acid | Pimelic acid | Meso-erythritol | ß-nocaryophillonic acid |
| 2016 | 0.2 | 0.2 | 0.7 | 1.2 |
| 2015 |  |  | 0.1 | 0.2 |
| 2014 |  | 0.2 | 0.4 | 0.9 |
| 2013 | 2.5 | 0.4 | 0.5 | 1.7 |
| 2012 | 0.2 | 0.2 | 0.5 | 0.9 |
| 2011 | 0.1 | 0.1 |  | 0.5 |
| 2010 | 0.2 | 0.0 |  | 0.1 |
| 2009 | 0.3 |  | 0.1 | 0.3 |
| 2008 | 0.3 |  | 0.1 | 0.3 |
| 2007 | 0.3 |  | 0.0 | 0.4 |
| 2006 |  |  | 0.1 | 0.2 |
| 2005 | 0.3 | 0.1 | 0.2 | 0.9 |
| 2004 | 0.6 |  |  | 0.2 |
| 2003 | 1.0 |  |  |  |
| 2002 | 0.1 |  | 0.4 |  |
| 2001 | 0.5 |  |  |  |
